# Chloroform Administration

**Published:** 1894-08

**Authors:** William Keiller

**Affiliations:** M. D., F. R. C. S., Ed., Professor of Anatomy, University of Texas; Late Chloroformist to Edinburgh Dental Hospital; Galveston


For Texas Medical Journal.

### CHLOROFORM ADMINISTRATION.

BY WILLIAM KEILLER, F. R. C. S., ED.,
Professor of Anatomy, University of Texas; Late Chloroformist to Edinburgh Dental Hospital.

TO THE SUPERFICIAL OBSERVER, at least, recent investigations and discussions on the subject of chloroform anæsthesia must have tended to induce a frame of mind comparable to Solomon's when he said "Vanity of vanities, all is vanity." So much learned testimony states that chloroform kills by cardiac paralysis, so much equally learned testimony declares that the respiration fails first; so many say it is almost criminal to use chloroform except in rare cases, so many state that properly administered it is equally safe with ether, and preferable in other respects.

In Britain, all the South of England swears by ether and condemns chloroform in the strongest terms; Scotland, and the North of England seldom employ anything else but the latter.

In America, the geographical distribution of opinion is reversed, the South favoring chloroform, the North being ether champions.

I do not propose to analyze the immense amount that has been written on either side of the controversy in recent years, but simply to call attention to the points which have seemed to me most important during a very considerable experience as a chloroformist. In private and hospital practice, I have used it constantly for surgical and obstetrical work, and as chloroformist to the Edinburgh Dental Hospital I acquired a pretty extensive experience in the use of chloroform as an anæsthetic. I do not have notes of my cases, so can only speak from careful general observation.

The equipment of the chloroformist cannot be too simple. The chloroform should be the purest the market can supply, and while Squibbs' may be no purer than that of other manufacturers, it has the reputation, is therefore likely to be scrupulously maintained up to the standard, and should an accident occur, it contributes to the physician's comfort to know that he used the best drug procurable.

A towel, rightly used, is the best inhaler, and all others are to be condemned, because unnecessary, troublesome to carry, and inconvient to keep clean. A towel and a pin are always at hand. But the method of covering up the face with a towel thrown over the hand, the hand making a mask over the patient's nose and mouth, cannot be condemned too strongly. The essentials of an inhaler are that it shall admit air with perfect freedom, afford a full view of the patient's face, and pour a constant, well-diluted stream of chloroform vapor down on the patient's nose and mouth. In face surgery, it is necessary that the inhaler should not occupy too much room. Select a small, pretty stiff towel (a handkerchief will not do), fold it, turning any fringes into the center, till it is nearly 10 by 4, then turn into a short, stiff cone, with a hole in the apex capable of admitting your thumb, and a base just large enough to include your patient's nose and mouth. Pour plenty of chloroform all round the upper half of the interior of the cone. You will thus avoid blistering your patient's face. When the cone is down over the face, the breath passes out and in through the hole in the apex, and the air is charged with chloroform from the sides of the cone as it is drawn in. Of inhalers, Esmarch's is the best for hospital use, but it is uncleanly, unless the flannel be regularly changed, as one cannot always prevent the patient spitting into the inhaler.

It is too clumsy to carry about to private cases. The simple flannel mask stretched on a small wire frame, from which a continuous stream of vapor is obtained by constant dropping from a bottle with a drop cork, is specially convenient in face operations, and gives a very constant vapor. The constancy of the amount of vapor given off is, however, of more theoretical than practical importance, and the towel, rightly folded, can be made as small and will be as little in the way as the wire mask. It should be remembered, of course, that chloroform vapor is heavier than air.

The anæsthetist should always, before starting, have prepared and placed within his own reach a hypodermic with 1-20 grain of strychnine. It is too late to prepare it when required. Of restoratives, I shall speak again by and by.

What about tongue forceps? Theoretically, there is no doubt that one can draw the tongue completely out of the way of the rima glottidi, by drawing forward the lower jaw, but it is always safe to have the tongue thoroughly under control. Ordinary tongue forceps apply too much pressure to the tongue, if left on, and it is good to have a tractor on the tongue, because there are certain cases of failure of respiration accompanied by muscular rigidity rendering it a matter of great difficulty to get hold of the tongue when it is wanted. There is always an objection to carrying a special instrument, or any instrument, if it can be done as easily without, so that I think the following suggestion, though appearing at first trivial, will recommend itself on experience of its simple efficiency. It occurred to me, a short time ago, to pass a large safety pin (of which there are usually plenty of new ones at any operation) through the tip of the tongue. It causes no irritation and leaves no pain behind it, is always at hand, and placed crosswise over the lips, keeps the tongue forward, or if the patient vomit, can be drawn lengthwise and let go so that he can swallow freely (which he cannot do unless the tongue be at liberty). It is well, perhaps, in addition to the above, to carry some nitrite of amyl capsules and three or four ounces of pure ether.

The safety of chloroform, as an anæsthetic, depends mainly on the mode of its administration. I believe I am right in saying, that it is in districts where ether is the main anæsthetic used that most chloroform deaths take place, and this I consider easily explicable, for there is such a difference in the forms of administration of the two drugs, that any one accustomed to the use of ether is thereby unfitted for using chloroform. The first rule in the administration of chloroform is, "begin gradually," and of all rules this is perhaps the most important. How often do we read in reports of cases of death from chloroform, that only a small quantity had been inhaled; and how often do patients, who have had chloroform before, plead with the physician when about to give it again, not to smother them. Both facts simply prove that again and again the anæsthetic is pushed too hard to begin with. I believe that a number of the deaths from heart failure arise from this cause. Let the physician push a saturated inhaler over his own nose and see how it feels. Add to the choking sensation which he experiences, the nervousness of the patient, nervousness which, but for his or her horizontal position, would be in many cases sufficient to cause syncope; and do you wonder that there are fatal cases of heart failure? Now this is all absolutely unnecessary. There is one case only with a sane patient where you have to push the chloroform from the first, and that is in the case of children where, I believe, it is absolutely safe. Get your patient's confidence, assure him that he wlll go to sleep quietly and peacefully, and administer it so that he never catches his breath. Above all things, don't be in a hurry. Hold the cone far from the nose to begin (chloroform vapor is heavy and pours like a stream of water down over the face), and your patient will come gradually under the influence, the reflexes will become less sensitive, and slowly you will get your cone down close over nose and mouth. Then you can push it; but if there be any catching of the breath, withdraw your towel for a moment. I believe that careful attention to this rule will save 30 per cent. of deaths from chloroform.

The next rule I would lay down is, be careful of the slightest obstruction to breathing. I do not like my patient to snore; I never let him "crow" twice if I can help it. We are tempted to disregard a little crowing and to think nothing of snoring at all. Now it is often the little things that decide between success and failure, life and death; and it is difficult to estimate how much even a very little interference with inspiration embarrasses the heart. There are certainly some patients whom it is difficult to prevent snoring, but in most cases a little change in the position of the head, turning it to the side, or lowering it and opening the mouth, etc., will stop this. Stertorous breathing may be due to spasm or paralysis of the laryngeal muscles.

If it be due to spasm (the crowing of early anæsthesia), remove the chloroform a little and draw forward the lower jaw or draw out the tongue if necessary. The crowing of paralysis is similarly treated; the point I would emphasize is, stop crowing at once; even the least trace of it is prejudicial to your patient.

Watch the respiration above all things; give it your undivided attention, train yourself to notice it even when you have to busy yourself about the other duties of your office as anæsthetist; and never divide your attention between the anæsthetic and the operation. You never know when respiration is to stop, and when it does so there is no sound, nothing marked to draw your attention. I can recall more than one case where I would probably have lost my patient had my attention been diverted from his respiration. Never forget that while you are giving chloroform your patient's life is in your hands; never get over-confident in your own experience as an anæsthetist; you have no time to laugh and joke while administering chloroform. It is most assuredly an unsafe drug in the hands of the careless. But surely doctors are never careless when performing such a duty! Perhaps not! Doctors are very human; and I have seen many anæsthetists I have also given chloroform often, and I know how easy it is to dream over a long operation when matters appear to be going smoothly. Remember the fallacy of watching chest and abdominal movements; they may go on most vigorously *with a closed glottis*, the result is cardiac strain, heart failure, and then you say your patient breathed several times after his heart stopped beating! The only infallible tests of respiration is to hear your patient breathing or to feel his breath with your hand. The hand held lightly over nose and mouth is an excellent and most convenient test when the patient is under the influence, and the towel removed. Train hand and ear to watch, and be ever on the alert; and again I say, beware of the smallest obstructions.

[ What about the pulse? That too must be watched; but don't stand with your fingers on the radial pulse and feel happy, and don't let your watch on the pulse interfere with your more important watch on the respiration. Note especially the condition of the lips and palpebral conjunctiva, before and during operation. A little paling of these will tell you almost as soon as the pulse of heart failure, or of vasomotor disturbance. This, with an occasional observation of the radial pulse, will keep sufficient note on the heart, and should lips pale and pulse grow weak and rapid, nothing will give a quicker result than 1-20 grain of strychnine. Your watch on lips and conjunctiva will also tell of respiratory insufficiency. A little blueness of the lips is a valuable warning.

How far is the anæsthetic to be pushed? It is generally agreed that the smallest operation requires full anæsthesia, and even for the drawing of a tooth or the incision of an abscess it should be pushed to abolition of the conjunctival reflex and muscular relaxation. Many deaths are reported for small operations where very little chloroform has been used. Such deaths are reflex, and the pulling of a tooth is more apt to produce fatal syncope if the patient be half under, than it would be when the patient is undrugged and fully conscious of the pain (itself a stimulant), or when he is thoroughly anæsthetized and reflexes are abolished. Anæsthesia once obtained is to be steadily maintained to the extent of complete relaxation of the voluntary (limb) muscles, and abolition of the conjunctival reflex (closure of the eyelids when the eyeball is touched with the finger). Probably the best guide is any tendency of the patient to shrink under the operator's hand. More chloroform has usually to be given for the introduction of the sutures. For abdominal and rectal operations the deepest anæsthesia is needed from first to last, as the abdominal muscles must in these cases be kept relaxed. Especially in rectal operations is deep anæsthesia necessary. For the extraction of the teeth I would, after much experience, advise deep anæsthesia that the jaw may be completely relaxed. Then, if I might presume to advise the dentist on the authority of the chief of the extracting department, when I was chloroformist in Edinburgh, I would say use no gag. With the jaw well relaxed, the finger and thumb of the left hand is the best gag and guide to the dental forceps as well. To the chloroformist, for dental operations I would say, do not be afraid of the semi-recumbency most convenient to the dentist, have your patient deeply under before you allow the dentist to start, abolish gags that your patient may be able to swallow blood which he cannot do freely with a gag in, stop the operation and give more chloroform if there be any sign of your patient coming to, and do not be afraid of blood getting into the larynx.

The condition of the pupil is a valuable indication of the depth of the narcosis. At first it is contracted, and it is seldom necessary to push the chloroform further than this contraction; next it dilates, and when the sphincter is thus paralyzed one of the last reflexes to yield is gone, and a little more chloroform means death. You may push your anæsthetic to this stage if necessary, but take care you don't go further.

It has never been my sad experience to have or to witness a death from chloroform, though, of course, I have had several cases in which respiration has stopped. First, there are cases where, in the early stages, the struggling passes into an epileptiform seizure which embarrasses so much both heart and respiration that the patient becomes rapidly cyanosed. I believe there is considerable danger in this condition. I feel sure that very gradual administration will diminish the tendency to these seizures, and I would especially emphasize the importance of beginning gradually in alcoholic cases. During the seizure it is best to continue the chloroform, to overcome the spasm of the respiratory muscles by artificial respiration, especially by intermittent pressure on the lower ribs, and I believe that in these cases of spasm, accompanied by cyanosis, nitrite of amyl is rapidly beneficial. In alcoholic patients a hypodermic of $\frac{1}{4}$ gr. morphine, with $\frac{1}{150}$ of atropine ten minutes before commencing the anæsthetic soothes the patient over. If you give morphine before administering chloroform, you must remember that you have done so and that *very little chloroform will then be required.*

Should the patient stop breathing, do not wait to see if it is only temporary, try pressure on the lower ribs, and if he does not breathe at once commence artificial respiration, slowly and collectedly, synchronous with your own breathing, beginning with a strong expiratory pressure to rid the trachea of chloroform vapor. See that the tongue is forward, and give 1-20 grain of strychnine, or rather get some one to give it, and to refill your syringe; but your chief duty is artificial respiration. I believe strychnine is the best cardiac and respiratory stimulant that can be used. Lowering the head, or inversion, nitrite of amyl if there be venous engorgement, electricity, hot and cold applications over the heart, forcible percussion over the heart, may all be valuable remedies; but these must all be strictly subservient to artificial respiration. I have had no experience of cardiac failure, respiration continuing; in abdominal operations one is liable to sudden attacks of pallor, and these should be met at once by strychnine given freely. In my hands, it has always had a rapid and marked influence. I consider it much better than ether, which I now never use hypodermically in a chloroform case. I believe that in abdominal operations the Trendelenburg position (the patient well elevated from the shoulders downward) is valuable in minimizing shock, and I would further suggets that the position, the legs from the knees downward being also elevated, might be used in primary operations for accidents where there has been much hemorrhage.

A word about vomiting. No post-mortem report of a case of death from chloroform is complete unless it be expressly stated that the trachea has been examined for vomited matters. In accident cases brought in drunk, in fact in all emergency cases, it would be a valuable precaution to wash out the stomach before administering the anæsthetic. A similar precaution might be valuable in operating for strangulated hernia. Should vomiting occur and the larynx be blocked, tracheotomy may be necessary. My colleague, Dr. Thompson, had a case where by a prompt tracheotomy he saved the patient's life.

The question may be asked, should the respiration fail and resuscitation be necessary before the operation is commenced, shall the operation be proceeded with?

I have repeatedly advised that it should proceed, and have never regretted it. Respiration does stop, for reasons that are not very definite, often before a knife has been used. A little strychnine, a few minutes artificial respiration, and all is as before. I have never had any hesitation in proceeding, and have not in these cases substituted ether for chloroform, nor have I ever had any further trouble. To defer the operation means to give some explanation to the patient, and has a bad moral effect on both patient and surgeon. Even were there marked cardiac failure, I would not be inclined to stop if the operation were necessary to life. Substituting ether, and giving cardiac stimulants, but never if possible letting the patient wake up, I would proceed as soon as the pulse and regular respiration would allow it. Were operation deferred, there would be as great a risk as ever of a repetion of the former experience.

I believe the main reasons why certain physicians may be liable to have accidents with chloroform are these: First, it is necessary to give ether in such concentration that any one accustomed to the use of ether will be extremely apt to give chloroform in the same way. To administer chloroform as one would ether would be death, and I feel sure that accidents happen by getting into the habit begotten of ether administration of giving the drug in too concentrated vapor. Another cause of trouble is impatience to get the patient under. There is nothing more dangerous than pushing chloroform too hard, especially at first, and great risk attends any attempt to commence the operation before the patient is fully under.

Too much anxiety about, and faith in the condition of the pulse tends to divert attention from the more important matter of the respiration; and there are cases where the pulse is reported to have failed first, but in which more careful attention to the breathing would have discovered that the cardiac failure was secondary to arrested respiration.

Lastly, I am convinced that one cause of failure to administer chloroform successfully is neglect of slight interference with the respiration. No interference, whether nasal or laryngeal, can be so slight that it is unimportant, and the slightest approach to crowing, or even snoring, should be at once attended to and relieved.

A word about one or two drugs used in conjunction with, or antagonistic to chloroform. While I was a hospital dresser with Mr. Joseph Bell in Edinburgh he was in the habit of using morphine hypodermically ten minutes before the operation in almost all his cases, and following his lead I have used it extensively, and believe it, if rightly used, a valuable adjunct to chloroform. A quarter of a grain of morphine with some atropine administered by hypodermic ten minutes before the anæsthetic, puts a nervous patient into a careless, less sensitive, more yielding frame of mind, and the chloroform is then taken quietly and easily. It has been said that there is danger in combining the drugs, and so there is decidedly, if the physican forgets that he has given morphine. After its administration one half or less of the chloroform will be sufficient that would have been required without it. Especially easy is it to keep such a patient under. Any one who has not tried the method will be astonished how little chloroform he requires, especially for a prolonged operation. I consider morphine used thus to be of great value with nervous patients, in prolonged operations, in abdominal cases where it is useful in quieting the intestines for a few hours, and in operations about the face where there may be some difficulty in administering the anæsthetic during the operation. I am not sure what effect the morphine has on chloroform nausea.

STRYCHNINE.—I am quite sure that there is no antagonist to chloroform so valuable as strychnine given in full doses and on the slightest sign of cardiac or respiratory failure.

Alcohol and ether rank far behind it. I always have 1-20 gr. in my hypodermic syringe before I commence, whether I use it or not, and it is the only stimulant I believe absolutely essential to the chloroformist's equipment. Caffeine and ammonia come next. Alcohol by enema is valuable later on to combat collapse.

ETHER.—Though advocating chloroform I cannot altogether pass over ether, though I have nothing new to say about it and no personal experience of its use. It is contra-indicated where it is difficult to administer the anæsthetic continuously as the patient recovers consciousness so quickly; thus it is not a good anæsthetic in face operations. It is inflammable and therefore must not be brought near an actual cautery. It is intensely irritating to the respiratory passages and apt to cause a large flow of bronchial secretion, hence it should not be used for old, feeble chronic bronchitics.

As a cardiac stimulant it is the best anæsthetic where the heart is fatty and feeble and there is no respiratory trouble, though my rule has been that where the patient is able to undergo an operation he is able to take chloroform, and I have seen no cause to alter my practice. Should there be symptoms of cardiac failure during the operation it is good to continue the anæsthesia with ether.

With regard to chloroform nausea, I think too little regard is paid to prophylaxis. In the case of sea sickness nothing is so important as treatment of the stomach before the voyage; and nothing will prevent chloroform sickness like careful dieting and some compound rhubarb powder during the three days preceding operation. In emergency cases washing out the stomach before operation would be most useful. To treat the nausea, absolutely nothing by the mouth, except perhaps a few drops of acidulated ice-cold water to relief the dryness of the mouth itself, and copious faintly saline enemeta to relieve thirst, though stern treatment from the patient's point of view, is the shortest and best road to relief. Ice is emphatically to be denied. An occasional teaspoonful of unsweetened ice-cold tea or coffee (without milk) may be allowed. In severe cases the stomach might be washed out with advantage.

I cannot dismiss the subject without referring to the necessity of teaching students how to give anæsthetics. Every doctor has to administer an anæsthetic frequently, yet the majority of students know nothing about it when they leave college. It should be the rule that each member of every class of ward assistants should act as chloroformist in turn, and that under responsible supervision, while one or two lectures and an examination on anæsthetics should be a matter of routine.

Further, as the country obstetrician or surgeon may have to trust to his nurse at any time to give the anæsthetic, all hospital-trained nurses should be taught to give chloroform and ether.

